# OFF-State-Specific Inhibition of the Proprotein Convertase Furin

**DOI:** 10.1021/acschembio.1c00411

**Published:** 2021-08-20

**Authors:** Sven O. Dahms, Tanja Haider, Gerhard Klebe, Torsten Steinmetzer, Hans Brandstetter

**Affiliations:** † Department of Biosciences, University of Salzburg, Hellbrunnerstraße 34, A-5020 Salzburg, Austria; ‡ Department of Pharmaceutical Chemistry, Philipps University Marburg, Marbacher Weg 6, D-35032 Marburg, Germany

## Abstract

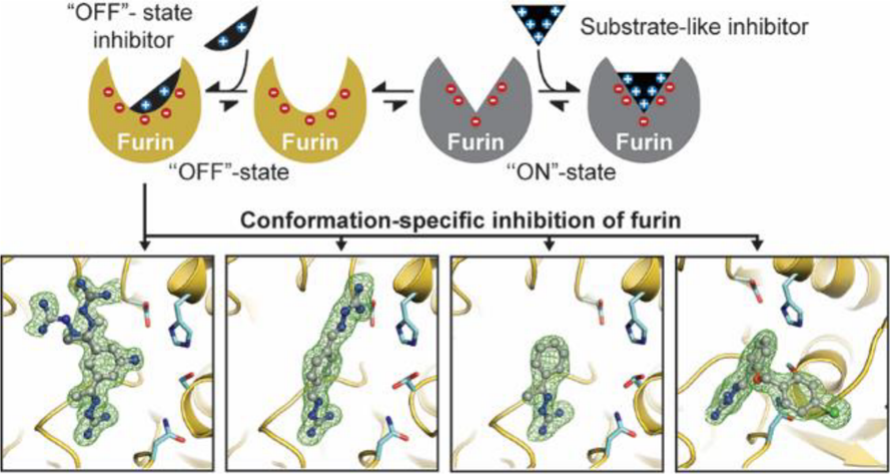

The pro-protein convertase furin is a highly specific serine protease involved in the proteolytic maturation of many proteins in the secretory pathway. It also activates surface proteins of many viruses including the severe acute respiratory syndrome coronavirus 2 (SARS-CoV-2). Furin inhibitors effectively suppress viral replication and thus are promising antiviral therapeutics with broad application potential. Polybasic substrate-like ligands typically trigger conformational changes shifting furin’s active site cleft from the OFF-state to the ON-state. Here, we solved the X-ray structures of furin in complex with four different arginine mimetic compounds with reduced basicity. These guanylhydrazone-based inhibitor complexes showed for the first time an active site-directed binding mode to furin’s OFF-state conformation. The compounds undergo unique interactions within the S1 pocket, largely different compared to substrate-like ligands. A second binding site was identified at the S4/S5 pocket of furin. Crystallography-based titration experiments confirmed the S1 site as the primary binding pocket. We also tested the proprotein convertases PC5/6 and PC7 for inhibition by guanylhydrazones and found an up to 7-fold lower potency for PC7. Interestingly, the observed differences in the *K*_i_ values correlated with the sequence conservation of the PCs at the allosteric sodium binding site. Therefore, OFF-state-specific targeting of furin can serve as a valuable strategy for structure-based development of PC-selective small-molecule inhibitors.

## Introduction

In addition to physiological protein maturation, furin activates the surface proteins of many human-pathogenic viruses,^1,2^ including the severe acute respiratory syndrome coronavirus 2 (SARS-Cov2).^3−5^ Acquisition of a furin cleavage site is regarded as a general pathogenicity factor for known viruses, and newly emerging viruses could also rely on this mechanism. Thus, furin inhibitors are regarded as promising antiviral therapeutics with a broad application potential.^6,7^

Furin belongs to the proprotein convertases (PCs), a family of serine proteases that share structural homology with the bacterial protease subtilisin. Largely different from subtilisin, the PCs are characterized by stringent substrate specificity. A subset of the PCs (PC1, PC2, Furin, PC4, PC5/6, PACE4, PC7) recognizes multibasic sequence motifs of the type (R/K)-X_*n*_-(R/K)↓ (*n* = 0, 2, 4; ↓ indicates the cleavage site; arginine is preferred at the P1 position).^8^ The typical consensus motif R–X–(R/K)–R↓^9,10^ has been utilized for the development of numerous substrate-like peptidic furin inhibitors (e.g., refs (11−19)). Structures of such inhibitors in complex with furin revealed typical side-chain interaction patterns at the P1, P2, and P4 positions driven by electrostatic interactions and extensive hydrogen-bond networks.^20^ Optimized substrate-mimicking inhibitors yielded very high potencies with *K*_i_ values in the low picomolar range.^21^ These compounds also revealed potent antiviral effects in cell cultures infected with several furin-dependent viruses and were well tolerated in mice and rats.^21^

**Figure 1:**
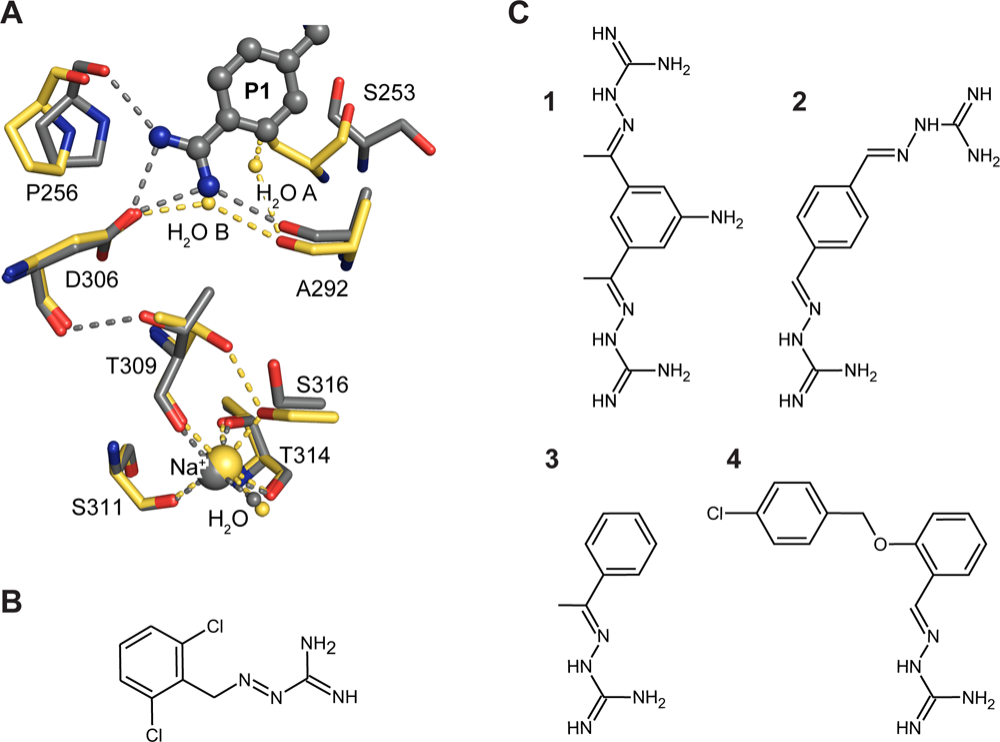
(A) Ligand binding to furin induces a conformational shift of furin from the ON- to the OFF-state. Superposition of furin in the ON- (gray, PDB ID: 5JXH(22)) and OFF-states (yellow, PDB ID: 5JXG(22)). Dashed lines highlight important interactions. Big and small spheres represent the sodium ion and water molecules, respectively. Instead of the P1 residue of the inhibitor, in the OFF-state a water molecule occupies two alternative sites (H_2_O A and B) at the bottom of the S1 pocket. (B) Chemical structure of guanabenz. (C) Chemical structures of the inhibitors used in this study.

Binding of substrate-like inhibitors to furin induces the substrate binding cleft to switch its conformation from the OFF-state to the ON-state.^22^ In the OFF-state, the alignment template region (also called the edge strand) between Ser253 and Pro256 adopts a specific conformation in which its typical beta-sheet-like interaction pattern with the substrate’s peptide backbone is prohibited. The S1 pocket is also partially blocked by Ser253 in the OFF-state (Figure 1A). Structural changes at furin’s S1 pocket are connected to an allosteric sodium-binding site. In the OFF-state, the sodium ion adopts an octahedral coordination sphere. Ligand binding induces a reconfiguration of hydrogen bonds, resulting in a change of the coordination sphere to tetragonal pyramidal in the ON-state (Figure 1A). The central players in this relay are the side chains of Thr309 and the Ser316, whereby the latter acts as sodium ligand in the OFF-state only. We concluded that the more stable octahedral coordination of the sodium binding site stabilizes the OFF-state.^22^ The gatekeeper function of the alignment template and especially Ser253 prevents the binding of monobasic peptides and is crucial for the stringent substrate specificity of furin. Capitalizing on this selection mechanism, we have proposed that specific targeting of the OFF-state might be utilized for the development of furin inhibitors.^22^

Several classes of nonpeptidic furin inhibitors have been reported in the literature.^23−27^ Small molecule inhibitors facilitate binding modes beyond the canonical substrate specificity pockets, as reported for 2,5-dideoxystreptamine derived compounds.^28^ Structural investigations revealed an unusual interaction of such inhibitors with the active site residues. Guanylhydrazone-derived compounds are less basic than guanidino or amidino compounds. For instance, the p*K*_a_ of the orally available antihypertensive guanylhydrazone-based drug guanabenz (Figure 1B) is 8.1 compared to 13 for guanidine.^29^ Another example is the anti-inflammatory guanylhydrazone Semapimod (also called CNI-1493, ref (30)), which reached phase 2 clinical trials. Recently, we showed that the reduction of the basicity of furin inhibitors resulted in increased bioavailability and reduced toxicity.^3,21^ Thus, guanylhydrazones are promising lead compounds for the development of next-generation furin inhibitors. However, the binding mode of these inhibitors is completely unknown so far.

In this study, we elucidated the structural basis of the interaction of guanylhydrazone inhibitors with furin. Our structural studies revealed characteristic binding patterns that require and stabilize the OFF-state conformation of the protease.

## Results and Discussion

### OFF-State-Specific Inhibition of Furin

Crystals of ligand-free furin usually grow under high salt conditions containing ∼3 M sodium chloride.^22^ Until now, attempts to investigate inhibitors with *K*_i_ values in the micromolar range or less, i.e., weaker inhibitors, by soaking constantly failed. We assumed that the high ionic strength shields furin’s highly charged active site cleft and competes with the electrostatic binding of ligands. Thus, we established new soaking conditions with reduced ionic strength. Supplementing sodium chloride with PEG8000 allowed us to reduce the sodium chloride concentration to 1 M at 10% PEG8000 or 0.25 M at 20% PEG8000. Crystals are stable over long time periods under the 1 M sodium chloride conditions, whereas the incubation time had to be reduced to ≤30 min at 0.25 M sodium chloride to prevent a decline of the diffraction power.

**Figure 2:**
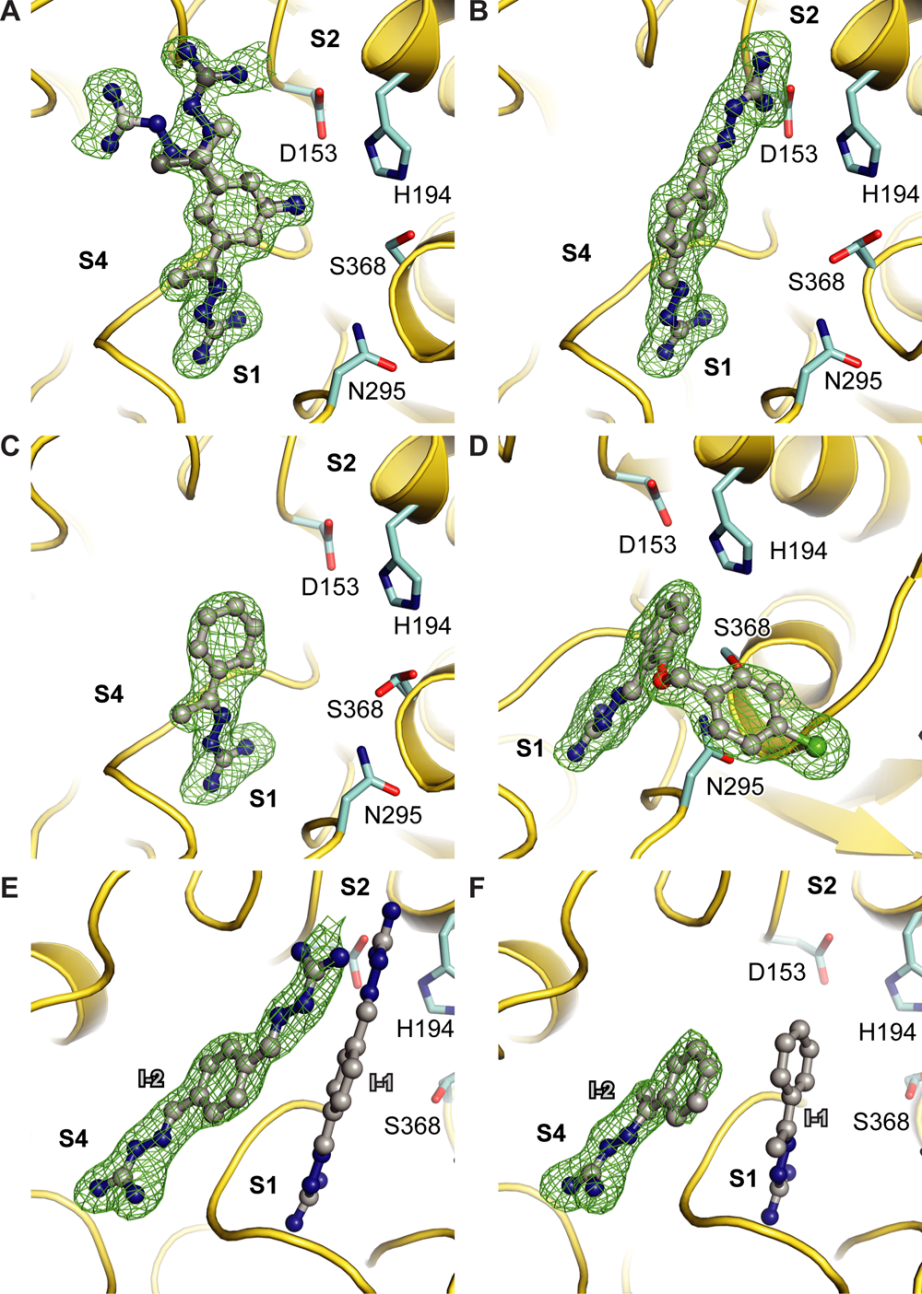
Structures of furin bound to guanylhydrazone-based inhibitors. The protease is shown as a cartoon representation (golden), catalytic residues as sticks with carbon atoms in cyan and the inhibitors as ball-and-stick models, respectively. The specificity pockets are labeled (S1–S4). The *F*_o_–*F*_c_ annealed omit electron density map of the inhibitors is shown as green mesh and is contoured at 3.0 σ. Furin in complex with inhibitors **1** (A), **2** (B), **3** (C), and **4** (D). Inhibitor **1** was found in two conformations shown with carbons in dark and light gray. (E,F) Two binding sites of **2** (E) and **3** (F) were found at the active site cleft of furin. The binding sites 1 and 2 are labeled I-1 and I-2, respectively.

**Figure 3:**
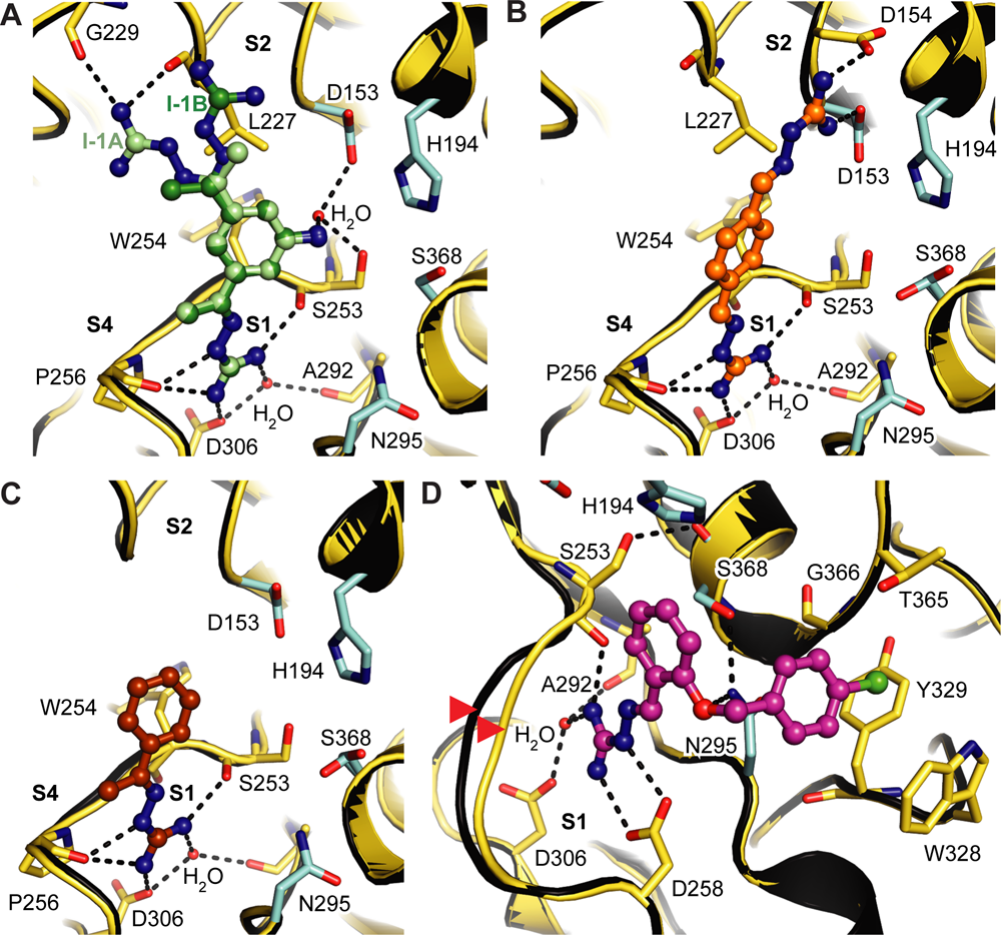
Binding mode of the OFF-state-specific furin inhibitors. Specific furin residues and the inhibitors are shown as stick and ball-and-stick models, respectively. Important interactions between the inhibitor and furin are marked with dashed lines. The furin backbone is given as a cartoon representation (yellow). The inhibitors **1** (A, two conformations are indicated by light and dark colors), **2** (B), **3** (C), and **4** (D) are shown with carbons in green, orange, brown, and magenta, respectively. The active site residues are shown with carbons in cyan. The specificity pockets are labeled (S1–S4). Ligand-free furin (PDB-ID: 5jxg(22)) is superimposed on the furin-inhibitor complex structures and is shown as a cartoon in black.

**Figure 4:**
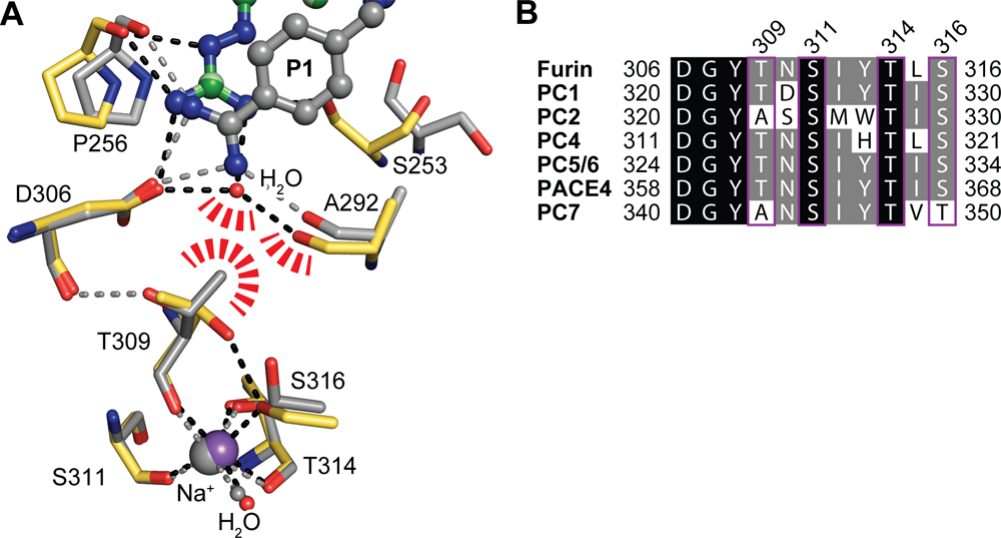
Allosteric connection between the S1 pocket and the sodium binding site. (A) Superposition of furin (carbons in yellow) bound to **1** (colored according to Figure 3A, OFF-state) and furin in the ON-state bound the substrate-like inhibitor 3-guanidinomethyl-phenylacetyl-RVR-4-aminomethyl-benzamidine (gray, PDB ID: 5jxh(22)). Competing space requirements of the water molecule at the S1 pocket and the side chain of Thr306 are highlighted by red dashes. Dashed lines highlight important interactions. For clarity, in the structure of furin bound to the substrate-like ligand, dashed lines, the Na^+^ ion and the H_2_O ligand are shown in gray. (B) Sequence alignment of the sodium-binding loop of furin-like PCs. Positions with sequence identities of 100% and >50% are highlighted in black and gray, respectively. Sodium binding residues are framed and numbered.

Using these newly developed soaking protocols, we investigated the structural basis of the interaction of four guanylhydrazones (Figure 1C) with furin. The structures of furin in complex with compounds **1** and **2** (Table 1, Figure 2A,B, stereo representation in Figure S1A,B) revealed a unique interaction mode of the inhibitors at the S1 pocket (Figure 3A,B). One of the guanylhydrazone moieties of the inhibitors specifically interacts with the alignment template of the protease (Figure 3A,B). This involves the carbonyl oxygens of Ser253 and Pro256 forming hydrogen bonds to the guanylhydrazone group. One of the terminal nitrogen atoms of the inhibitor’s guanylhydrazone moiety also mediates an electrostatic contact with Asp306. The other terminal nitrogen atom mediates a hydrogen bond to a specific water molecule at the S1 pocket of furin. This water molecule is bound to the enzyme by hydrogen bonds to the side-chain carboxylate group of Asp306 and to the carbonyl oxygen of Ala292 (Figure 3A,B). Taken together, this is a largely different interaction network than observed for substrate-like inhibitors requiring the ON-state of the protease (Figure 4A, Figure S2A). The P1 residue of a substrate-like inhibitor inserts more deeply into the S1 pocket, and thereby a nitrogen atom of the amidine expels and replaces the water molecule. In the ON-state, the peptide bond between Ser253 and Trp254 is also rotated by 90°, uncovering the S1 pocket and allowing β sheet-like interactions with the ligand backbone at the same time.^22^ Other residues that are involved in the OFF-state to ON-state transition also adopt a typical OFF-state conformation with **1** and **2** bound (Figure S3A,B). This includes the sodium-binding site showing the OFF-state-specific coordination sphere (Figure S4A,B). The side chain of Ser316 contributes to the octahedral coordination of sodium. By contrast, in the substrate-bound ON-state, the hydroxyl group of Ser316 flipped 120° away from the sodium ion adopting a tetragonal pyramidal coordination (Figure 4A). The reorientation of Ser316 also prevents its interaction with Thr309. Thus, in the ON-state, Thr309 mediates a hydrogen bond with the carbonyl oxygen of Asp306 instead. The side-chain flip of Thr309 in the OFF-state also provides more space on the bottom of the S1 pocket and thus facilitates binding of the water molecule to Asp306 and Ala292 (Figure 4A). In the ON-state orientation of Thr309, however, its methyl group would clash with this water molecule. The carbonyl oxygen of Ala292 also moves by 0.7 Å toward the S1 pocket to avoid a clash with the methyl group of Thr309 in the ON-state.

**Table 1:** Data Collection and Refinement Statistics

inhibitor	**1**	**2**	**2**	**3**	**4**
**data collection statistics**					
PDB ID	7O1U	7O1W	7O1Y	7O20	7O22
soaking concentration (mM)	20	20	40	100	40
wavelength (Å)	0.9184				
space group	*P*6_5_22				
unit cell parameters: *a* = *b* (Å), *c* (Å)	131.7, 155.7	131.6, 156.0	131.1, 156.5	130.3, 156.0	131.0, 155.5
resolution range^a^ (Å)	47.2–1.7 (1.80–1.70)	47.3–1.8 (1.91–1.80)	47.4–1.7 (1.80–1.70)	47.2–1.8 (1.91–1.80)	47.1–1.8 (1.91–1.80)
*R*_meas_^a^ (%)	14.8 (187.9)	14.8 (187.9)	11.2 (193.1)	16.5 (276.3)	19.1 (345.0)
I/sigI^a^	18.7 (2.0)	16.6 (1.7)	15.1 (1.3)	16.3 (1.2)	15.2 (1.1)
CC_1/2_ (%),^44^^,^^a^	99.9 (79.1)	99.9 (75.6)	99.9 (61.2)	99.9 (60.4)	99.9 (55.0)
completeness^a^	98.7 (97.6)	99.1 (98.0)	99.1 (98.5)	98.5 (97.0)	99.6 (99.0)
no. of observations (total/unique)	1 734 173/86 732	1 457 796/73 502	668 083/86 405	1 434 222/71 888	1 458 226/72 880
**refinement statistics**					
no. of non-hydrogen atoms	4375	4335	4321	4190	4130
protein/inhibitor/water/other	3776/42/519/80	3816/54/424/95	3732/54/475/114	3718/52/376/96	3646/21/427/57
Rwork/Rfree	15.6/16.7	16.1/17.9	16.3/17.7	16.3/17.6	17.0/18.3
B-factors (Å^2^)					
overall/Wilson plot	28.8/26.2	31.2/29.4	31.4/29.5	35.1/33.3	35.1/33.5
protein/inhibitor/water/other	24.9/26.6/35.0/32.3	28.4/32.6/36.5/36.4	28.1/32.4/38.5/37.6	32.5/32.2/39.2/38.6	33.5/31.5/39.9/40.2
RMSD bond length (Å)	0.005	0.009	0.009	0.009	0.006
RMSD bonded B-factors (Å^2^)	3.3	3.4	3.7	2.6	2.3

a Values of the highest resolution shell are given in parentheses

The guanylhydrazone moieties of the inhibitors **1** and **2** exhibit meta- and para configurations, respectively. The second meta-substituted guanylhydrazone moiety of **1** is found in two different, nearly equally occupied conformations (Figure 3A, Table S1). In conformation A (Figure 3A, light green carbons), it contacts the carbonyl oxygens of Gly229 and Leu227. In conformation B (Figure 3A, dark green carbons), the elongated guanylhydrazone moiety of **1** more efficiently covers the hydrophobic surface of the side-chain of Leu227. The second guanylhydrazone moiety increases the positive net charge, which is in general favorable at the highly negatively charged binding site. The aniline-like amino group of **1** interacts only indirectly through water-mediated hydrogen bonds with the side chains of Ser253 and Asp153. Inhibitor **1** also contains an additional methyl group at the guanylhydrazone moiety, and the rather flat molecule efficiently covers the hydrophobic surface at the rim of furin’s S1 pocket.

**Figure 5:**
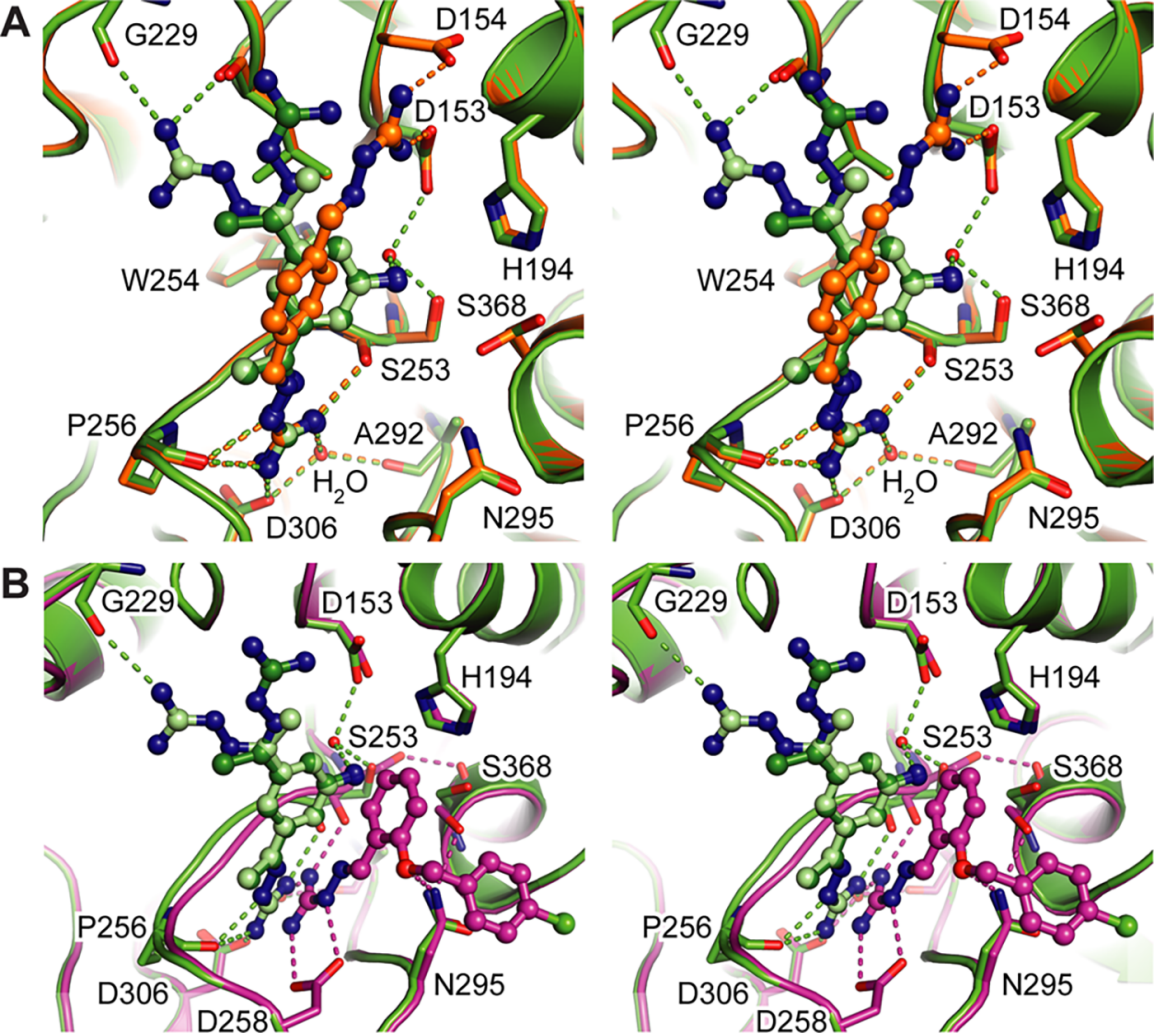
Comparison of the binding poses of guanylhydrazones. Stereo representations of structural superpositions of furin-inhibitor complex structures. Specific furin residues and the inhibitors are shown as stick and ball-and-stick models, respectively. Important interactions between the inhibitor and furin are marked with dashed lines. The inhibitors are colored according to Figure 3. Furin residues and the furin backbone are colored according to the inhibitors in green (**1**), orange (**2**), and magenta (**4**). (A) Superposition of furin in complex with **1** and **2**. (B) Superposition of furin in complex with **1** and **4**.

The more elongated para configuration of **2** facilitates charge-assisted hydrogen bonds to the side chains of Asp153 and Asp154. It should be noted that Asp153 is part of the catalytic triad and Asp154 usually contributes to S2–P2 interactions with substrate-like inhibitors. To mediate these additional interactions, the central phenyl ring and the second guanylhydrazone moiety of **2** are rotated by ∼60° compared to **1** (Figure 5A). In this orientation, inhibitor **2** mediates less hydrophobic contacts with furin compared to inhibitor **1**. The more extensive hydrogen-bond pattern of **2** in comparison to **1** might thus be compensated by weaker hydrophobic interactions. This observation could explain the similar *K*_i_ values for furin by inhibitors **1** and **2** of 3.3 μM and 3.1 μM, respectively (Table 2).

**Table 2:** *K*_i_ of Inhibitors **1** and **2** for Selected PCs

inhibitor	furin [μM]	PC5/6 [μM]	PC7 [μM]
**1**	3.3 ± 0.1	3.6 ± 0.2	22.4 ± 1.9
**2**	3.1 ± 0.2	1.7 ± 0.2	10.4 ± 0.7

According to our structural data, a minimal OFF-state-specific binder should comprise a phenyl group with a single guanylhydrazone substituent. To prove this assumption, we solved the structure of furin in complex with compound **3** (Table 1, Figure 2C, stereo representation in Figure S1C). Indeed, the structure revealed a largely similar S1-binding pattern as observed for inhibitors **1** and **2** (Figure 3C). For compound **3**, a *K*_i_ value of 273 μM has been reported.^26^ The largely reduced potency is well explained by the missing interactions of the second guanylhydrazone group, which is lacking in **3**.

### Involvement of the S1′ Pocket in Alternative OFF-State-Specific Binding by Inhibitor **4**

Inhibitor **4** (Figure 1C), initially published by Komiyama and co-workers, contains a single guanylhydrazone substituent.^24^ Different from derivative **3**, however, the reported *K*_i_ values of 11.8 μM^24^ or 25.3 μM^26^ revealed a 1 order of magnitude stronger furin inhibition. In **4**, an additional *para*-chlorobenzyloxy substituent is attached in the ortho position to the guanylhydrazone arm. According to the binding modes observed for **1**–**3**, we were expecting that the ortho-attachment of **4** would result in steric clashes at the S1 pocket. Therefore, we assumed that **4** must adopt a different binding pose compared to the other inhibitors. To verify this hypothesis, we solved the structure of furin in complex with analogue **4** (Table 1, Figure 2D, stereo representation in Figure S1D). Here, the guanylhydrazone moiety is tilted by ∼40° compared to **1** toward the catalytic serine (Figure 3D, Figure 5B). The direct interactions with Asp306 and Pro256 are lost and instead substituted by a salt bridge to the carboxyl group of Asp258. The hydrogen-bond to the carbonyl oxygen of Ser253, however, is maintained as well as the water-mediated interaction to the carbonyl oxygen of Ala292 and the carboxylate group of Asp306 (Figure 3D, Figure S3D). This is facilitated by a displacement of the alignment template, resulting in a relocation of Ser253 by 1.3 Å (based on Cα) and a rotation of its side chain by 120°. The side-chain flip of Ser253 enables a hydrogen bond to the carbonyl oxygen of the catalytic Ser368 (Figure 3D). We also observed a hydrogen-bond between the amide nitrogen of Asn295 from furin’s oxy-anion hole and the oxygen of the *p*-chlorobenzyl ether moiety of inhibitor **4**. The chlorophenyl ring inserts into a small hydrophobic pocket surrounded by Gly366, Tyr329, Trp328, and Asn295, the putative S1′ site of furin (Figure 3D). The relocations of the alignment template and catalytic residues induced by compound **4** impressively demonstrate the structural plasticity of furin’s active site cleft.

### Second Binding Site of Inhibitor **2** and **3** at the Substrate Binding Cleft of Furin

**Figure 6:**
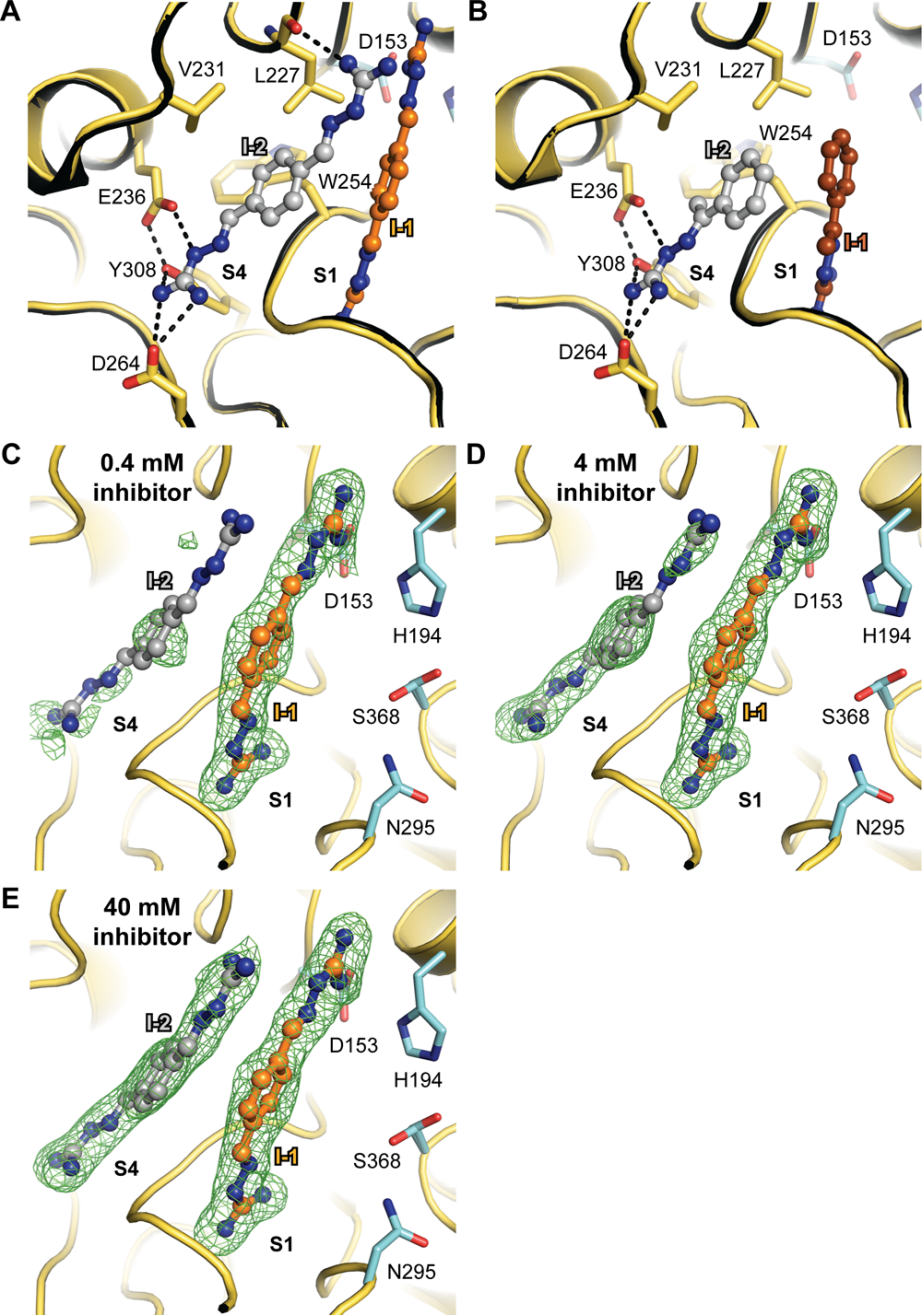
Second binding site of **2** and **3**. (A–E) The furin backbone is given as a cartoon representation (yellow). Specific furin residues and the inhibitors are shown as stick and ball-and-stick models, respectively. The active site residues are shown with carbons in cyan. The specificity pockets are labeled (S1, S4). (A, B) The second molecule of **2** (A) or **3** (B) is shown with gray carbons (I-2). Inhibitor molecules bound to the S1 pocket are colored according to Figure 1 and Figure 2. Important interactions between the inhibitor and furin are marked with dashed lines. Ligand-free furin (PDB-ID: 5jxg(22)) is superimposed and is shown as a cartoon in black. (C–E) Titration of furin at low ionic strength (250 mM NaCl) with 0.4 mM (C), 4 mM (D), and 40 mM (E) of inhibitor **2**. The *F*_o_–*F*_c_ annealed omit electron density map of the inhibitors is shown as a green mesh and is contoured at 3.0 σ.

Interestingly, we found a second binding site of **2** and **3** at furin’s substrate binding cleft involving residues of the S4 pocket (Figure 6A,B, Figure S5A, C). This second binding site revealed similar occupancies of the inhibitors but increased B-factors compared to the binding site at the S1 pocket (Table S1). One guanylhydrazone group mediates salt bridges to Glu236 and Asp264 as well as a hydrogen bond to Tyr308. The phenyl ring covers the hydrophobic stretch build up by Val231, Leu227, Trp254, and Gly255. The inhibitor molecule bound at the S1 pocket further extends this hydrophobic surface. The second guanylhydrazone group of **2**, which is bound to the S4 pocket, is also aligned to the other molecule at S1 (Figure 6A). In addition, it mediates a hydrogen bond to the carbonyl oxygen of Leu227. Superposition of furin in complex with inhibitor **2** and a substrate-like inhibitor revealed different salt bridge configurations with Asp264 (Figure S6A). P4–arginine interacts in an end-on bidentate configuration with Asp264. In complex with inhibitor **2**, the Cα of Asp264 is displaced by 0.6 Å away from the S4 pocket. Thus, only one carboxyl oxygen atom remains in interaction distance with the guanylhydrazone moiety. This orientation of Asp264 was also observed in the structure of furin in complex with inhibitor **1** (Figure S6B,C) and in ligand free furin.^22^ We conclude that this effect might be a specific OFF-state feature rather than a ligand dependent effect at the S4 pocket.

Because of the lower number of specific interactions, we assumed that the binding affinity of **2** for the S4 pocket is lower as compared to the S1 pocket. To prove this hypothesis, we titrated crystals with increasing inhibitor concentrations. The strength of the electron density directly correlates to the occupation of the binding sites with the inhibitor. Indeed, we found the S1 pocket already occupied by **2** at 0.4 mM as indicated by the occupancy of 75% from crystallographic refinement (Table S2). In contrast, very weak electron density of this compound was observed at S4 (Figure 6C). A partial occupation of 63% by **2** at S4 was observed at a 4 mM concentration (Figure 6D) and an occupation of 82% was reached at 40 mM (Figure 6E). The refined occupancy of inhibitor **2** at the S1 pocket at 40 mM concentration was 0.94. It should be noted that water molecules occupy the substrate binding pockets in absence of any ligand.^22^ The binding sites of these water molecules overlap with the binding sites of the inhibitors and affect crystallographic occupancy refinement. Thus, the refined occupation should always be considered in context with the electron density (Figure 6C–E). Nonetheless, these results show that **2** binds much tighter at S1 compared to S4. We also observed a third binding site of **2** and **3** outside of the substrate binding cleft. Here, two inhibitor molecules are stacked between two arginine residues of a crystal contact. This binding mode is specific for the crystal lattice and thus is not expected to occur in solution (Figure S5B, D).

On the basis of the inhibitor titration experiments, we estimate the apparent dissociation constant of **2** at the S4 binding site in the low millimolar range. Because of the very similar binding mode, a similar affinity can be expected for **3**. Thus, it is tempting to speculate that the chemical merging of **2** and **3** to target the S1, S2, and S4 pockets simultaneously would result in a more potent inhibitor. This strategy might increase the affinity of follow-up compounds up to several orders of magnitude.

### OFF-State-Specific Inhibitors As Structural Probes for PCs

Due to a lack of structural information, it is not yet clear whether ligand binding also induces an ON-to-OFF-state transition in other PCs. On the basis of sequence alignments, the residues that participate in the conformational transition at the S1 pocket are conserved in all PCs.^31^ This includes the residues involved in the direct interaction with the inhibitors **1** and **2**. In previous studies, we could show that the structural differences between the OFF- and ON-states also involve allosteric sites in furin.^22^ The S1 pocket and the Na^+^-binding site seem to be connected to allow an efficient cross-talk between both sites (Figure 4A). The central players of this communication line are Thr309 and Ser316 (see above). For all herein investigated inhibitors, furin adopted the octahedral OFF-state coordination of the sodium-binding site (Figure S3A–D and Figure S4A–D). Therefore, we concluded that the more stable octahedral Na^+^ coordination sphere supposedly stabilizes the OFF-state.^22^ Interestingly, the crucial amino acids Thr309 and Ser316 are replaced by Ala and Thr in PC7 (Figure 4B). If the stability of the OFF-state is affected by this relay, one would expect an influence on binding of the guanylhydrazone inhibitors and specifically a reduction of their affinity to PC7. To test this hypothesis, we determined the *K*_i_ values of inhibitors **1** and **2** for PC7 and used furin as well as PC5/6 as control proteins. The sodium binding residues, including Thr309 and Ser316, are identical in furin and PC5/6 (Figure 4B). Thus, we would expect similar affinities of both inhibitors for these PCs. Indeed, inhibitor **1** was less potent for PC7 as indicated by a ∼7-fold potency loss of the *K*_i_ (Table 2). While inhibitor **1** interacts almost exclusively with the S1 pocket, inhibitor **2** also interacts with Asp153 and Asp154 in the S2 pocket. The conformation of these residues is not OFF-state-specific. In conclusion, we would expect a less pronounced effect on the *K*_i_ of compound **2**. In excellent agreement with this hypothesis, we observed only a ∼3-fold increase of the *K*_i_ of inhibitor **2** for PC7 compared with furin (Table 2). In contrast, **1** and **2** showed similar *K*_i_ values for PC5/6 and furin. Inhibitor **2** was even more potent for PC5/6 compared to furin, which might indicate that the interaction of **2** with Asp153 and Asp154 contributes more strongly for this protease. Taken together, our data suggest that PC7 is capable of adopting the OFF-state conformation, although it seems to be less stable compared to furin and PC5/6. These data also emphasize the importance of the sodium-binding site and its influence on ligand binding by the different PCs. Although the amino acids of the S1 pocket are 100% conserved among the PC family members, their conformation could be influenced by an allosteric effect of distant sites, as shown for the sodium-binding site. Because these amino acids are less conserved, conformation-specific inhibitors could be more selective for certain PC family members. Thus, OFF-state-specific interactions could be utilized to increase the specificity of next generation furin inhibitors.

## Methods

### X-ray Crystallography

Equal volumes of homogeneously glycosylated human furin (ref (20), ∼10 mg mL^–1^ in 10 mM Hepes, pH 7.5, 100 mM NaCl, 2 mM CaCl_2_) and crystallization solution (100 mM MES, 200 mM K/NaH_2_PO_4_, pH 5.5–6.0, and 2 M NaCl) were mixed and equilibrated against the reservoir (3.0–3.2 M NaCl) in vapor diffusion experiments at 18–20 °C as described previously.^32,22^

The optimized soaking solutions contained either 1.0 M NaCl, 200 mM Mes/NaOH, at pH 5.5, 10% (w/v) PEG8000, and 20% (v/v) DMSO or 0.25 M NaCl, 200 mM Mes/NaOH, at pH 5.5, 20% (w/v) PEG8000, and 20% (v/v) DMSO and were supplemented with the specific inhibitors. Inhibitors **1** and **2** were soaked at 20 mM concentration in 1 M NaCl containing soaking solution for 0.5 h. Inhibitor **3** and **4** were soaked in 0.25 M NaCl containing soaking solution for 0.5 h at an inhibitor concentration of 100 mM and 20 mM, respectively. For the inhibitor titration experiments with **2**, 40 mM, 4 mM, or 0.4 mM of the inhibitor was soaked for 0.5 h in 0.25 M NaCl-containing soaking solution. Soaked crystals were flash cooled in liquid N_2_. Diffraction data collection was performed at the synchrotron beamline BL14.2^33^ (BESSY-II) of Helmholtz-Zentrum Berlin (HZB). The data were processed using XDS^34^ with XDS-APP^35^ (v2.0) and the CCP4 program suite^36^ (v.7.1.001). COOT^37^ (v.0.8.9.2) was used for model building. Refinement was performed in PHENIX^38^ (v1.18.2) using the PDB ID 5JXG(22) as an initial model. One specific R_free_-set (initially generated up to 1.0 Å, ref (22)) was transferred to the data sets prior refinement start. Geometry restraints of the inhibitors were obtained from the PRODRG server^39^ and from the GRADE Web server (http://grade.globalphasing.org). All guanylhydrazones were modeled in the E configuration, which is in agreement with previous studies with model compounds.^40^ Electron density omit maps were calculated in PHENIX^38^ (v1.18.2). PYMOL was used for molecular graphics (http://www.pymol.org) and structural alignments. In the case of inhibitor titration experiments with **2**, the structure was first built and refined for a crystal soaked at 40 mM. This structure was used to calculate refinements with (for occupancy calculation) and without (for omit map calculation) an inhibitor in the model for three additional crystals independently soaked at 40 mM, 4 mM, and 0.4 mM (Table S2) obtaining *R*/*R*_free_-values of 17.3/19.0, 17.5/19.1, and 17.7/19.3, respectively. The occupancy refinement yielded consistent B-factors between the three differently soaked structures (Table S2). Structure factors and coordinates of the complexes of furin with inhibitors **1**, **2**, at 1 M NaCl; **2** at 0.25 M NaCl; and **3** and **4** have been deposited as PDB entries 7O1U, 7O1W, 7O1Y, 7O20, and 7O22, respectively.

### Protein Expression and Purification

HEK293S cells were stably transfected with the coding sequences of human furin,^20^ PC5/6 (Arg33-Gly634, UNP^41^ Q92824) and PC7 (Pro44-Thr667, UNP^41^ Q16549) cloned into the pCDNA3.1(+) vector (ThermoFisher). Cells were grown in DMEM (4.5 g L^–1^ glucose, 2 mM l-glutamine, 110 mg l^–1^ sodium pyruvate, 3.7 g L^–1^ NaHCO_3_, nonessential amino acids) supplemented with 10% (v/v) FBS. Polyclonal selection of stable cell lines was conducted in the presence of 500 μg mL^–1^ G418 (Geneticin). The cells were grown in multilayer flasks (HyperFlask M, Corning) without G418 for large-scale protein expression. The medium of confluent expression cultures was exchanged with DMEM supplemented with 2 mM sodium butyrate as well as with FBS (2% (v/v), furin) or without FBS (PC5/6 and PC7). The medium exchange was repeated five (furin) or two (PC5/6 and PC7) times, and the centrifuged medium (20 min, 5500 g, 4 °C) was stored at −80 °C until further use.

The purification of human furin from a conditioned medium has been shown previously.^20^ PC5/6 and PC7 were purified by immobilized metal affinity chromatography and gel permeation chromatography (GPC), as shown for furin but excluding the inhibitor affinity purification step.^20^ PC7 (50 μg mL^–1^) was fully activated in 50 mM Tris/HCl, at pH 7.5, 10 mM CaCl_2_, and 0.15 M NaCl, at pH 7.5, in the presence of 2.0 μg mL^–1^ thermolysin for 16 h at 37 °C. To remove thermolysin, the reaction mixture was concentrated in an ultrafiltration device (30 kDa cutoff, amicon, Millipore) and subjected to GPC (both steps at 4 °C). Purified PC5/6 and PC7 were stored at −20 °C.

### Enzyme Kinetics

Enzyme kinetic measurements were performed in 100 mM HEPES/NaOH, at pH 7.0, 2 mM CaCl_2_, and 0.2% (v/v) Triton X 100 with the fluorogenic substrate pyr-ERTKR-7-amino-4-methylcoumarin (Bachem) at 37 °C. The 100 μL reactions were prepared in 96-well black half-area plates (Corning), preincubated for 15 min at 37 °C and measured in a 96-well plate reader (Tecan infinite 200) at excitation and emission wavelengths of 380 and 460 nm. Because of the autofluorescence of inhibitor **1**, the *K*_i_ values were determined at constant inhibitor concentrations (**1**: 20 μM for furin, 15 μM for PC5/6 and 50 μM for PC7; **2**: 10 μM for furin, 5 μM for PC5/6 and 15 μM for PC7) and 1:2 dilution series of the substrate (Figure S7). The *K*_M_ values were determined with 1:2 dilution series of the substrate in the absence of an inhibitor (4.7 ± 0.4 μM for furin, 2.5 ± 0.2 μM for PC5/6, and 19.3 ± 0.8 μM for PC7; Figure S7). Enzyme kinetic data were evaluated with GRAPHPAD PRISM (GraphPad Software) using the prebuild “Michaelis–Menten” model to calculate *K*_M_ and a competitive inhibition model (*v* = *V*_max_ × *S*/[*S* + *K*_M_(1 + *I*/*K*_i_)]; *v* = reaction velocity, *V*_max_ = maximum velocity, *S* = substrate concentration, *K*_M_ = Michaelis–Menten constant, *K*_i_ = inhibition constant) for *K*_i_ determination. All measurements were performed in triplicate.

### Sequence Alignments

Sequence alignments of the Uniprot^41^ entries for furin (P09958), PC1 (P29120), PC2 (P16519), PC4 (Q6UW60), PC5/6 (Q92824), PACE4 (P29122), and PC7 (Q16549) were calculated with EMBL-EBI Clustal Omega^42^ and modified with the ALINE software.^43^

## Supporting Information Available

Supporting Figures S1–S7 as well as Supporting Tables S1 and S2 (PDF)
