# Supplementary material for: OFF-State-Specific Inhibition of the Proprotein Convertase Furin
Source: ACS Chem Biol. 2021 Aug 20;16(9):1692–700. doi: 10.1021/acschembio.1c00411 (PMC8453481; doi:10.1021/acschembio.1c00411)
Supplement: Supplementary file 1 — cb1c00411_si_001.pdf [file cb1c00411_si_001.pdf]

Supporting Information

OFF-state-specific inhibition of the proprotein convertase furin.

Sven O. Dahms[1,*], Tanja Haider[1], Gerhard Klebe[2], Torsten Steinmetzer[2] and Hans Brandstetter[1]

[1] Department of Biosciences, University of Salzburg, Hellbrunnerstraße 34, A-5020 Salzburg, Austria

[2] Department of Pharmaceutical Chemistry, Philipps University Marburg, Marbacher Weg 6, D-35032 Marburg, Germany

* Corresponding author e-mail address: sven.dahms@sbg.ac.at

Table of contents

	Page
Supporting Figures	
Figure S1	S2-S3
Figure S2	S4
Figure S3	S5-6
Figure S4	S7-8
Figure S5	S9-10
Figure S6	S11
Figure S7	S12-13
Supporting Tables	
Table S1	S14
Table S2	S15
Supplementary References	S16

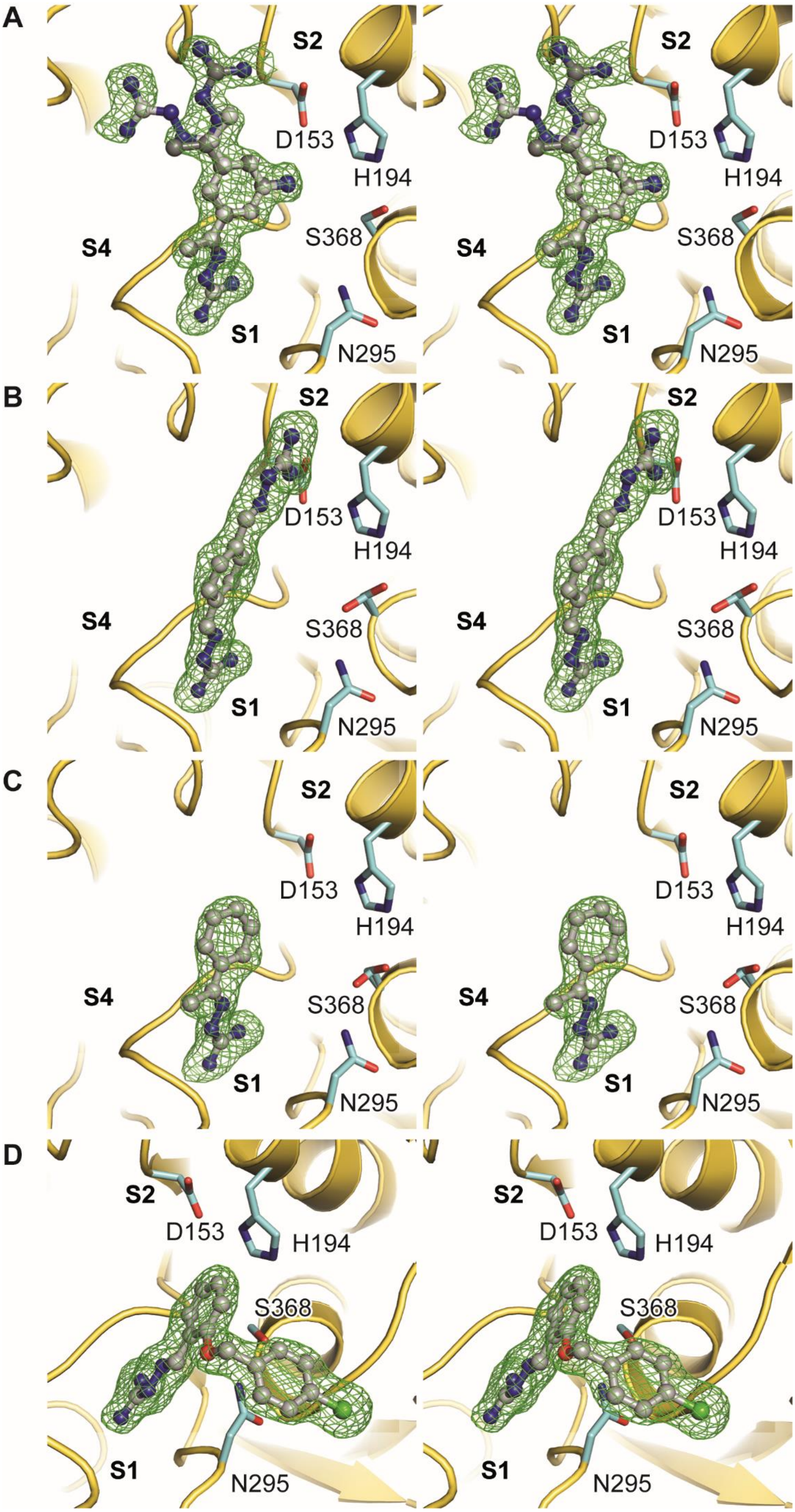
A
S2
D153
H194
S368
S4
S1
N295
B
S2
D153
H194
S368
S4
S1
N295
C
S2
D153
H194
S368
S4
S1
N295
D
S2
D153
H194
S368
S1
N295

Figure S1 Stereo view of the substrate binding cleft of furin with bound inhibitors **1** (A), **2** (B), **3** (C) and **4** (D). The protease is shown as cartoon representation (golden), catalytic residues as sticks with carbon atoms in cyan, and the inhibitors as ball-and-stick models, respectively. The $\boldsymbol{F_o}$-$\boldsymbol{F_c}$ annealed omit electron density map of the inhibitors is shown as green mesh and is contoured at 3.0 σ.

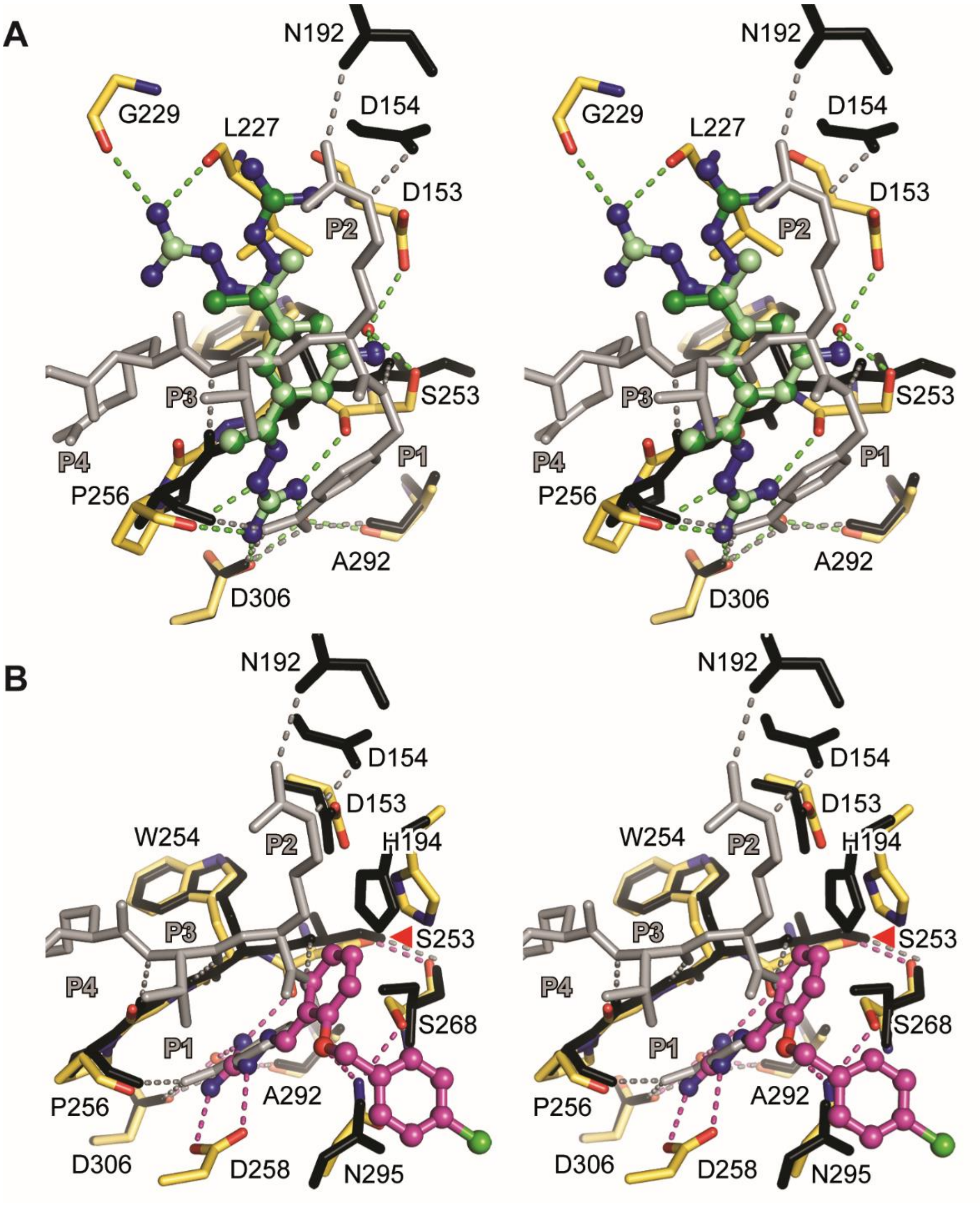

Figure S2. Comparison of the binding modes of OFF-state inhibitors and substrate-like (ON-state) inhibitors. Stereo representations of superposed structures. Specific furin residues and the inhibitors are shown as stick and ball-and-stick models, respectively. Important interactions between the inhibitor and furin are marked with dashed lines colored according to the inhibitors. Residues of furin (yellow carbons) bound to OFF-state inhibitors (colored according to Figure 3, the inhibitors **1** and **4** are shown with carbons in green and magenta, respectively) are given as stick and ball-and-stick model, respectively. Furin (black stick model) bound to the substrate-like inhibitor 3-guanidinomethyl-phenylacetyl-RVR-4-aminomethyl-benzamidine (grey stick model, PDB-ID: 5jxh[1]) was superposed with furin in complex with **1** (A) or **4** (B).

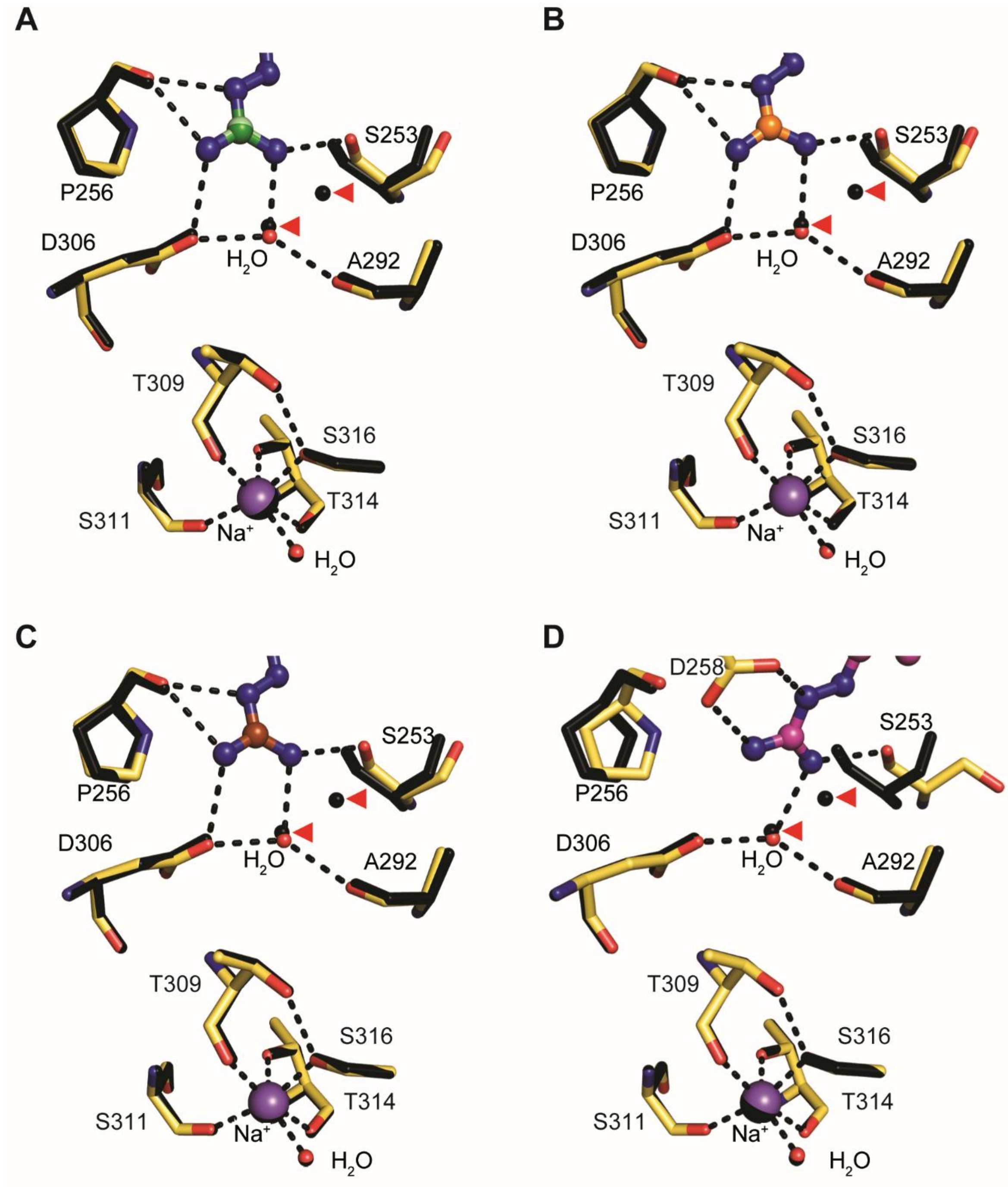

Figure S3 Allosteric connection of the S1-binding site and the sodium binding site. Residues of inhibitor-bound furin are shown as stick model and the inhibitors **1** (A, two conformations, light green and dark green carbons), **2** (B, orange carbons), **3** (C, brown carbons) and **4** (D, magenta carbons). Dashed lines highlight important interactions. For comparison, unliganded furin (PDB-ID: 5jxg) was aligned with the inhibitor-bound structures and is shown as cartoon in black. A specific water molecule bound to Asp306 and A292 contributes to interactions

with the inhibitor. This water molecule occupies two alternate sites in ligand free furin and is highlighted by red arrowheads.

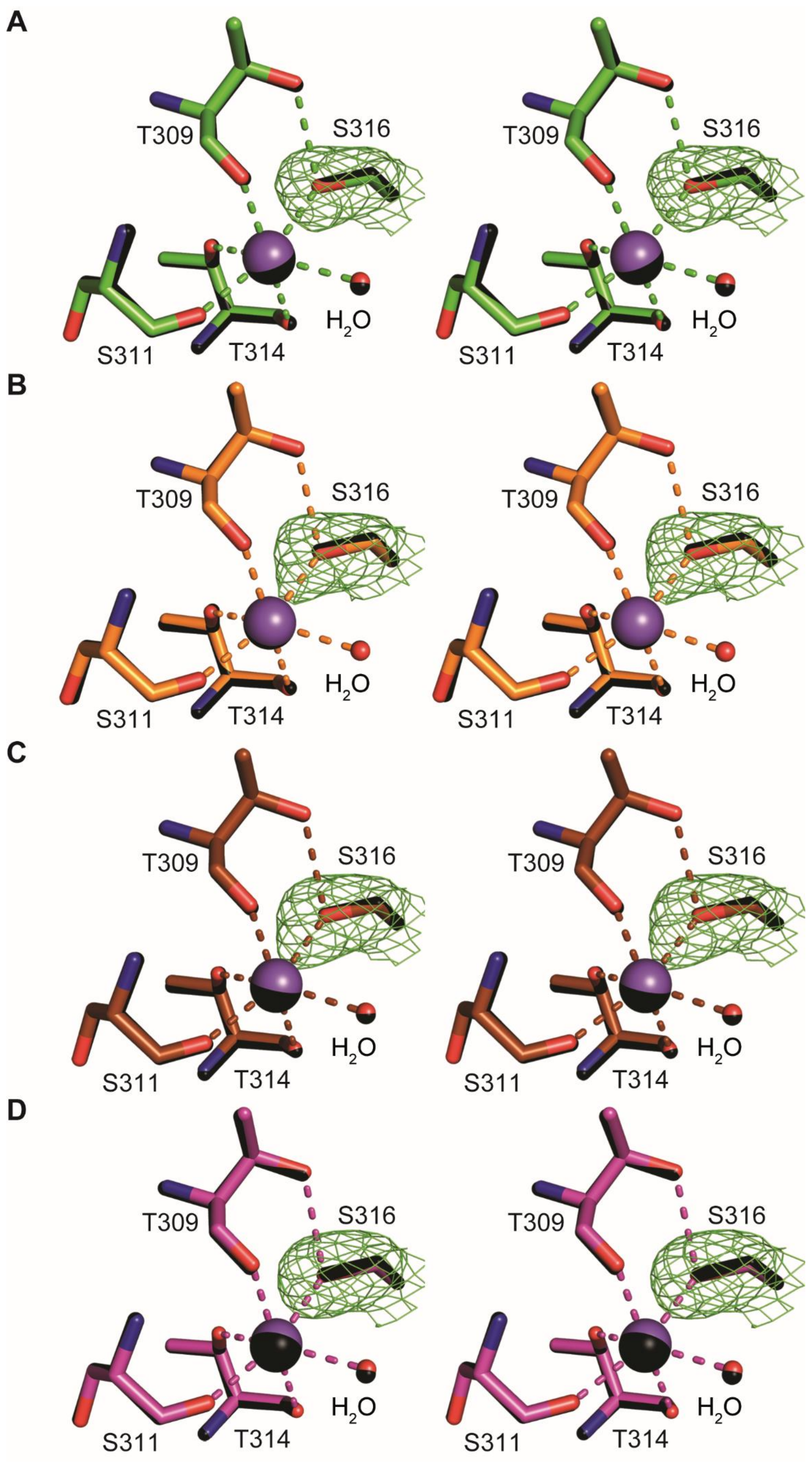
A
T309
S316
H$_2$O
S311
T314
B
T309
S316
H$_2$O
S311
T314
C
T309
S316
H$_2$O
S311
T314
D
T309
S316
H$_2$O
S311
T314

Figure S4 Close view of the sodium binding cleft of furin with bound inhibitors **1** (A), **2** (B), **3** (C) and **4** (D). The sodium binding residues and the sodium ion are shown as stick model and purple sphere, respectively. Important interactions are highlighted with dashes. For comparison, unliganded furin (PDB-ID: 5jxg[1]) was aligned with the inhibitor structures and is shown in black. The $\boldsymbol{F_o}$-$\boldsymbol{F_c}$ annealed omit electron density map of S316 is shown as green mesh and is contoured at 5.0 σ.

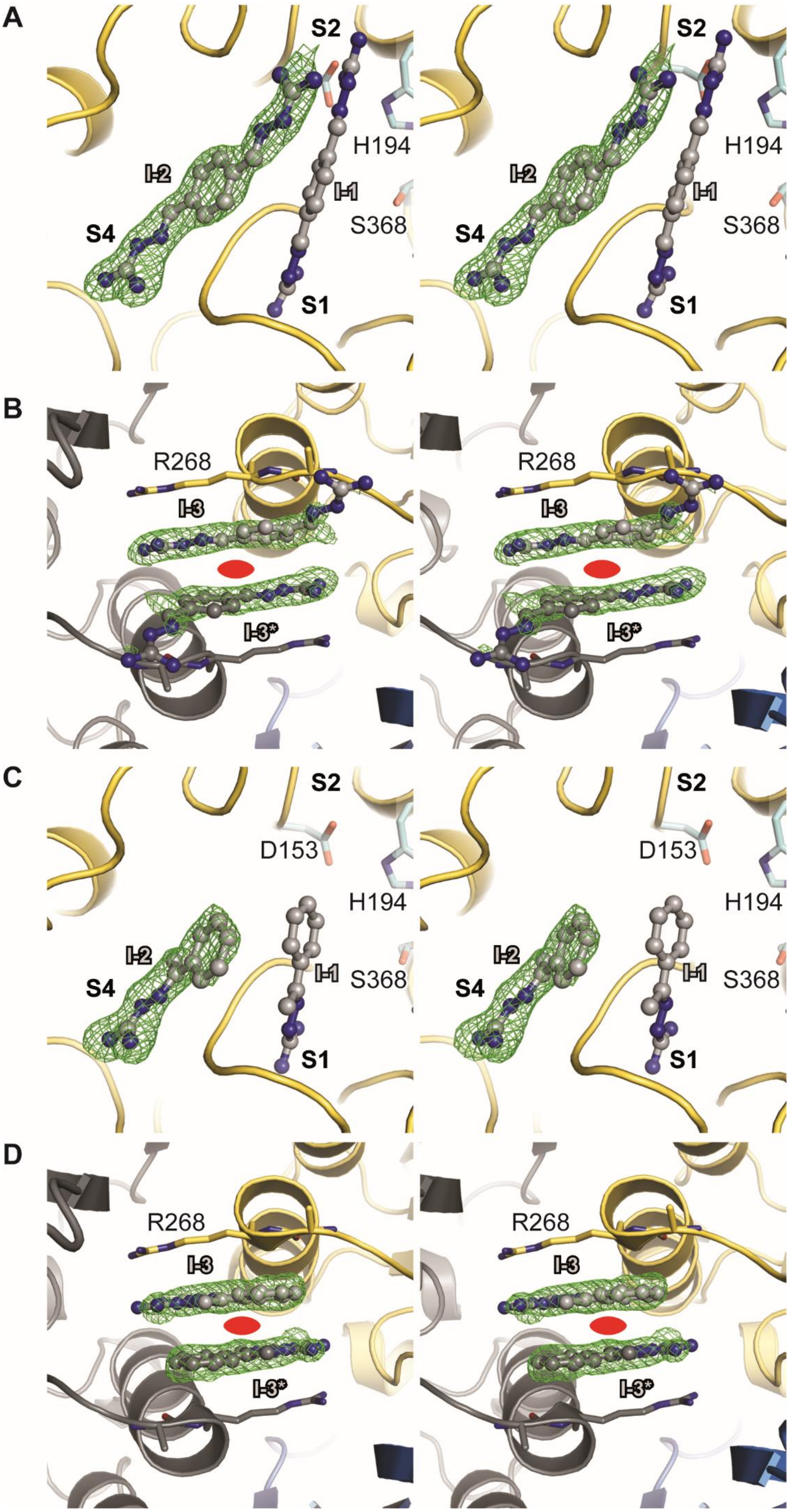

A
S2
H194
I-2
I-1
S368
S4
S1
S2
H194
I-2
I-1
S368
S4
S1
B
R268
I-3
I-3*
R268
I-3
I-3*
C
S2
D153
H194
I-2
I-1
S368
S4
S1
S2
D153
H194
I-2
I-1
S368
S4
S1
D
R268
I-3
I-3*
R268
I-3
I-3*

Figure S5 Additional binding sites of **2** (A, B) and **3** (C, D). Furin is shown as cartoon representation (golden), catalytic residues as sticks with carbon atoms in cyan, and the inhibitors as ball-and-stick models (light grey), respectively. The F_o-F_c annealed omit electron density map of the inhibitors is shown as green mesh and is contoured at 3.0 σ. (B, D) A third inhibitor molecule of **2** and **3** is bound at a crystal contact on a two-fold symmetry axis (marked in red). The symmetry mates of furin (cartoon and sticks) and of the inhibitor molecule (I-3*) are shown in dark grey.

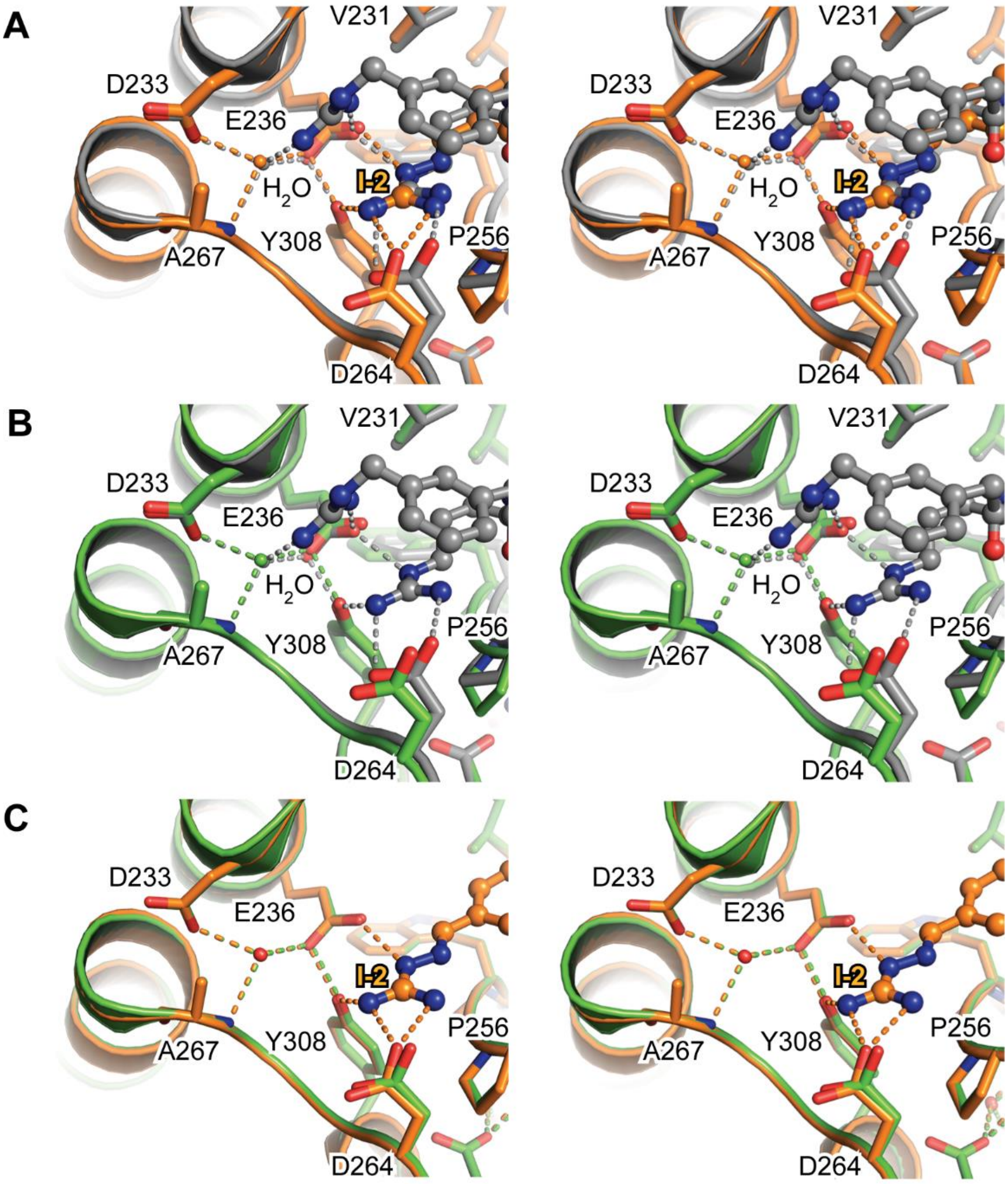

Figure S6. Impact of ligand binding on the conformation of furin's S4/S5-pocket. Stereo representations of structural superpositions of furin-inhibitor complex structures. Specific furin residues and the inhibitors are shown as stick and ball-and-stick models, respectively. Inhibitors, furin residues and the furin backbone are colored in green (**1**) orange (**2**) and grey (guanidinomethyl-phenylacetyl-RVR-4-aminomethyl-benzamidine, PDB-ID: 5jxh). Important interactions between the inhibitor and furin are marked with dashed lines in the colors of the respective inhibitors. (A) Superposition of furin in complex with phenylacetyl-RVR-4-aminomethyl-benzamidine (ON-state) and (A) **2** or (B) **1**. (C) Superposition of furin in complex with **1** and **2**.

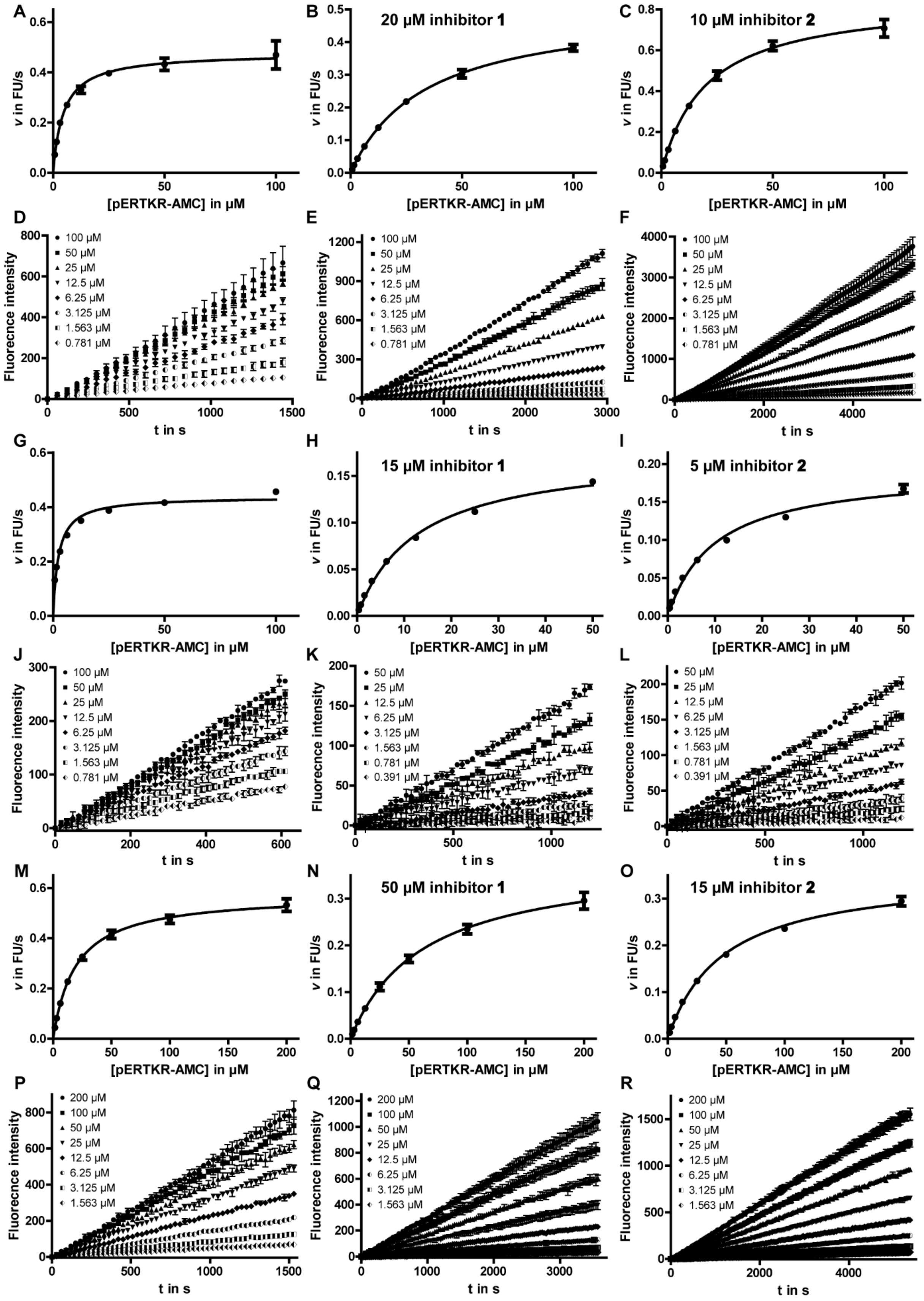

Figure S7. Curve fits and progress curves of enzyme kinetic analyses. Mean values and standard deviations of three measurements are shown. (A) K_M and (B-C) K_i determinations

with Furin and respective raw data (D-F) of different substrate concentrations (µM). (G) K_M and (H-I) K_i determinations with PC5/6 and respective raw data (J-L) of different substrate concentrations (µM). (M) K_M and (N-O) K_i determinations with PC7 and respective raw data (P-R) of different substrate concentrations (µM).

Table S1. Occupancy refinement of inhibitors.

**Inhibitor**	**1**		**2**			**2**			**3**				**4**
PDB ID	7O1U		7O1W			7O1Y			7O20				7O22
Soaking concentration (mM)	20		20			40			100				40
Molecule #	1		1	2	3	1	2	3	1	2	3		1
Specificity pocket	S1		S1	S4	-	S1	S4	-	S1	S4	-		S1/S1’
Alternate conformation	A	B	-	-	-	-	-	-	-	-	A	B	-
Occupancy	0.47	0.53	0.94	0.84	0.72	0.95	0.87	0.74	0.74	0.71	0.44	0.56	1.00
Average B-factor (Å^2)	26.8	26.4	25.2	38.0	34.6	23.7	34.3	39.2	29.5	32.5	33.5	33.4	31.5

Table S2. Data collection statistics and refined occupancies of crystals titrated with inhibitor **2**

**Data collection statistics**									
Concentration of **2** (mM)	**40**			**4**			**0.4**		
Wavelength	0.9184			0.9184			0.9184		
Space group	$P6_522$			$P6_522$			$P6_522$		
Unit cell parameters: a = b (Å), c (Å)	130.6, 156.0			131.4, 155.4			131.0, 155.6		
Resolution range [a] (Å)	47.2-1.9 (2.01-1.90)			47.2-1.9 (2.01-1.90)			47.2-1.9 (2.01-1.90)		
R_{meas} [a] (%)	25.5 (324.9)			27.7 (375.3)			26.5 (369.4)		
I/sigI [a]	12.6 (1.2)			12.6 (1.0)			11.8 (1.0)		
$CC_{1/2}$ (%) [a]	99.8 (60.1)			99.8 (48.8)			99.8 (51.9)		
Completeness [a]	99.6 (99.0)			99 (97.9)			99.3 (98.4)		
No. of observations (total/unique)	1235293 / 62032			1236671 / 61886			1233543 / 62242		
Wilson B-factor	34.9			35.7			36.5		
**Refined occupancies**									
Molecule #	1	2	3	1	2	3	1	2	3
Specificity pocket	S1	S4	-	S1	S4	-	S1	S4	-
Occupancy	0.94	0.82	0.66	0.87	0.63	0.38*	0.75	0.39*	0.35*
Average B-factor (Å^2)	26.4	37.0	41.9	27.4	38.0	42.9	28.8	39.3	44.2

[a] Values of the highest resolution shell are given in parentheses, * Very weak electron density

References

[1] Dahms, S. O., Arciniega, M., Steinmetzer, T., Huber, R., and Than, M. E. (2016) Structure of the unliganded form of the proprotein convertase furin suggests activation by a substrate-induced mechanism, *Proceedings of the National Academy of Sciences of the United States of America 113*, 11196-11201.
